# A series of PDB-related databanks for everyday needs

**DOI:** 10.1093/nar/gku1028

**Published:** 2014-10-28

**Authors:** Wouter G. Touw, Coos Baakman, Jon Black, Tim A. H. te Beek, E. Krieger, Robbie P. Joosten, Gert Vriend

**Affiliations:** 1 Centre for Molecular and Biomolecular Informatics, CMBI, Radboud university medical center, Geert Grooteplein Zuid 26–28 6525 GA Nijmegen, The Netherlands; 2 Bio-Prodict BV, Nieuwe Marktstraat 54E, 6511 AA Nijmegen, The Netherlands; 3 Department of Biochemistry, Netherlands Cancer Institute, Plesmanlaan 121, Amsterdam 1066 CX, The Netherlands

## Abstract

We present a series of databanks (http://swift.cmbi.ru.nl/gv/facilities/) that hold information that is computationally derived from Protein Data Bank (PDB) entries and that might augment macromolecular structure studies. These derived databanks run parallel to the PDB, i.e. they have one entry per PDB entry. Several of the well-established databanks such as HSSP, PDBREPORT and PDB_REDO have been updated and/or improved. The software that creates the DSSP databank, for example, has been rewritten to better cope with π-helices. A large number of databanks have been added to aid computational structural biology; some examples are lists of residues that make crystal contacts, lists of contacting residues using a series of contact definitions or lists of residue accessibilities. PDB files are not the optimal presentation of the underlying data for many studies. We therefore made a series of databanks that hold PDB files in an easier to use or more consistent representation. The BDB databank holds X-ray PDB files with consistently represented B-factors. We also added several visualization tools to aid the users of our databanks.

## INTRODUCTION

The Protein Data Bank (PDB) is the worldwide repository of macromolecular structures determined experimentally mainly by X-ray crystallography, NMR or electron microscopy (1–4). The more than 100.000 entries in the PDB form a valuable source of information, and PDB entries are used all over the world in a wide variety of research projects in academia and industry alike.

When the PDB was conceived in 1971 (5), the initiators could hardly have imagined that their punchcard based PDB file format (1,6) would survive for more than 40 years. Although the PDB has superseded its archaic punchcard compromise between inclusiveness and human readability by the PDBx/mmCIF file format (3,6), in practice the old format is still used by most software applications. The PDB format is the source of many problems, some of which are addressed by our databanks.

All databanks are available from http://swift.cmbi.ru.nl/gv/facilities/. This site also provides extensive documentation that includes help for downloading individual files or whole databanks.

## UPDATE ON EXISTING DATABANKS

Table 1 lists the main databanks with a brief description of their content. In the following sections we will describe the progress on the existing systems since we previously reported on them (7).

**Table 1.** List of databanks, their content and location

**Existing databanks**	
DSSP	Secondary structure of proteins http://swift.cmbi.ru.nl/gv/dssp/
HSSP	Multiple sequence alignments of UniProtKB against PDB http://swift.cmbi.ru.nl/gv/hssp/
PDBFINDER and PDBFINDER2	Searchable PDB entry meta-data and derived information http://swift.cmbi.ru.nl/gv/pdbfinder/
PDBREPORT	Lists many types of anomalies and errors in structures http://swift.cmbi.ru.nl/gv/pdbreport/
PDB_REDO	Re-refined and rebuilt crystallographic structure models http://www.cmbi.ru.nl/pdb_redo/
PDB_SELECT	Quality-sorted sequence-unique PDB chains http://swift.cmbi.ru.nl/gv/select/
WHY_NOT	Explains why entries in any databank do not exist http://www.cmbi.ru.nl/WHY_NOT/
**New databanks**	
BDB	PDB entries with a consistent B-factor representation http://www.cmbi.ru.nl/bdb/
WHAT IF Lists	Lists for protein structure bioinformaticians http://swift.cmbi.ru.nl/gv/lists/
YASARA Scenes	YASARA scenes showing protein structure properties http://www.cmbi.ru.nl/pdb-vis/

Existing databanks were published earlier (7).

Most of our systems have been prepared for the PDB's transition from the PDB file format to the mmCIF file format. Most databanks are now also available derived from PDB_REDO structure models.

DSSP is the *de facto* standard for the assignment of secondary structure elements in PDB entries. The DSSP (8) software has been rewritten to better recognize π-helices (8–10). The determination of π-helices still follows the original description by Kabsch and Sander (8), but the assignment of π-helices is now given precedence over the assignment of α-helices, which should prevent underestimating the number of π-helices (9,10).

HSSP (11–15) multiple sequence alignments (MSAs) are now created with an improved version of the original Sander and Schneider (11) algorithm. These files are available in the original HSSP format and in Pfam Stockholm format (16). The Stockholm format is used by applications like HMMER (17) and Jalview (18). Projects like BioJava (19), BioPerl (20) and Biopython (21) provide parsers for these Stockholm-formatted files.

The PDBFINDER and PDBFINDER2 databanks (22) are now created using a slightly modified algorithm that better deals with exceptions in PDB files. The changes to the DSSP and HSSP software have also been taken into account. Furthermore, the two single flat text files are now compiled from separate files for each PDB ID.

New developments in the PDB_REDO decision-making algorithms were described elsewhere (23). Many recent improvements in PDB_REDO focus on enabling user-friendly data-mining and visualization. A list of all significant structural changes like changed rotamers and flipped peptide planes (24) is available in an easy-to-mine format to quickly figure out whether PDB_REDO has changed residues-of-interest in a particular PDB entry. Model validation data such as WHAT_CHECK Z-scores, crystallographic R-factors, per-residue measures of fit to the crystallographic data (real-space R-factors (25), and real-space correlation coefficients (26)), as well as comprehensive descriptions of ligand quality and structural interactions (27) are now also provided. A description of all data from PDB_REDO entries is given in Supplementary Table S1.

PDB_SELECT (28) now also provides quality-sorted sequence-redundant lists. These lists do not include entries deemed unwanted for bioinformatics purposes, e.g. entries that contain too many severe errors, too many incomplete or non-canonical amino acids or homology models.

The WHY_NOT indexing algorithm has been adapted to deal with the many novel databanks.

## A NEW MAJOR DATABANK

Macromolecules are not static. The displacement of atoms in crystal structures can be modeled at various levels of detail. B-factors are commonly used to model the displacement of single atoms, while translation, libration and screw-rotation (TLS) parameters model the displacement of groups of atoms. Unfortunately, the meaning of the B-factor values on the ATOM records of PDB files is not always unambiguous. For example, ‘residual’ rather than ‘full’ B-factors have been reported for thousands of PDB structure models for which both B-factors and TLS parameters had been refined. Residual B-factors do not include the contribution of the TLS motion (29). The Databank of PDB files with consistent B-factors (BDB) (30) homogenizes the B-factor representations in PDB files to aid the bioinformatics and protein engineering applications that depend on B-factors (e.g. (31–36)). For every crystallographic PDB entry there is a BDB entry. The files in the BDB are simply identical to those in the PDB if full B-factors have been reported, but they contain full B-factors calculated from the PDB file data if the meta-data in the PDB file suggest that this is necessary.

## WHAT GOOD IS BEAUTY, IF IT IS NOT TO BE SEEN?

The main users of PDB files are bioscientists in fields as diverse as drug design, molecular biology or biofuel engineering. These researchers often are not aware of all problems that come with the use of PDB files; see, for example, the B-factor problems that we addressed with the BDB.

**Figure 1.**
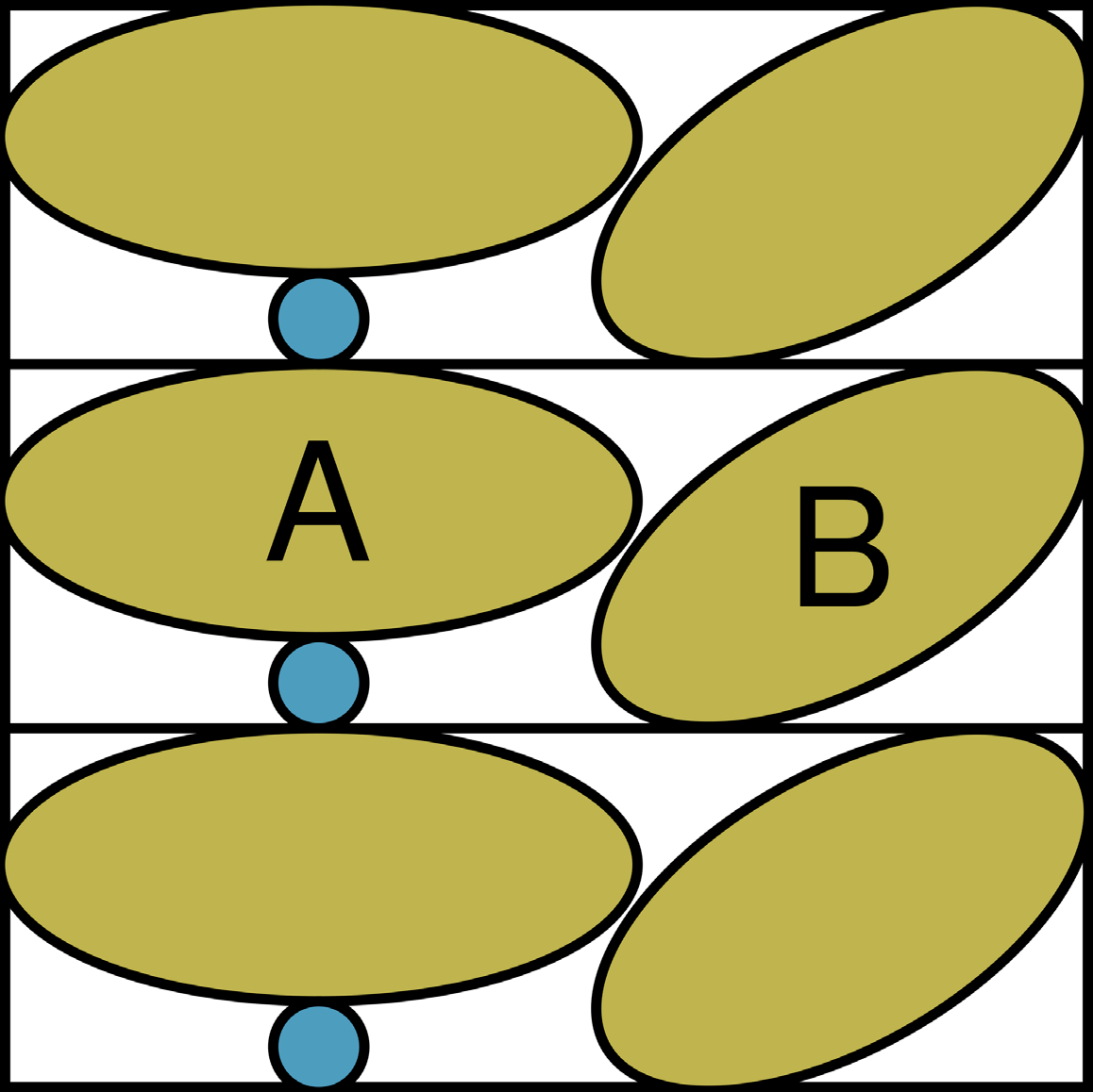
Schematic illustration of the absence of symmetry related waters, ions and ligands. Three crystal cells are shown. These three cells, obviously, contain the same molecules. The brown ellipses A and B are two macromolecules, and the small blue circle is a small molecule, e.g. water, that sits packed between two copies of macromolecule A. Each macromolecule A thus has a contact with two small blue circles. The PDB file corresponding to this example will only contain the content of one cell. So when the PDB file is inspected visually, only one of the blue circles will be seen.

A problem that is ubiquitous for all structures solved by X-ray crystallography is the implicit description of symmetry related ions, waters and ligands (the absence of symmetry related macromolecules has been solved, for example, with PISA (37). Figure 1 illustrates this problem.

The problem illustrated in Figure 1 has been addressed in two databanks. One databank holds PDB files that include the symmetry-related waters. A second databank has been compiled from PDB files with a shell of symmetry-related residues.

Solvent exposed amino acid side chains tend to be mobile, and as a consequence are not observable in the electron density map that is at the basis of modeling atomic coordinates in X-ray crystallography. The absence of side chains is unlikely to remain unnoticed, but may cause problems for protein structure software or perturb structural analyses otherwise. We therefore made one databank in which we computationally filled in the missing side chains using the rotamer library that is also at the basis of WHAT IF's homology modeling module (38).

## DATABANKS SPECIFICALLY FOR BIOINFORMATICIANS

Any aspiring protein structure bioinformatician will need to write or obtain a PDB file parser before he or she can start working on the intended research project. Writing a parser that can cope with a large enough fraction of all problems in PDB files can be a major practical problem. We made a large number of databanks to overcome this problem in many cases. These databanks include the per-residue molecular and solvent accessible surface area, the secondary structure in four states (helix, strand, turn, loop), the number of crystal contacts, torsion angles and backbone angles. Lists of salt bridges and metal-coordinating residues are also created.

Recently, several groups made breakthroughs in the field of *ab initio* protein structure prediction (39–41). The idea behind these methods is that correlations between the variability patterns of residue positions i and j in a multiple sequence alignment are indicative for a contact between those residues i and j. We believe that these studies could benefit from a better definition of what constitutes an inter amino acid contact. To support research in this field a large group of databanks has been made in which contacting amino acids are listed. In each databank contacts are defined in a different way (direct atomic contacts; Cα–Cα distances; side chain contacts only; etc.).

## NEW VISUALIZATION TOOLS

The CMBI databanks provide a wealth of structure-related information. We aim to provide this information in files that are bioinformatician-friendly. Not all users, however, may feel equally comfortable writing scripts to show the number of crystal contacts per residue in 3D, to create entropy-variability (EV) plots from an HSSP alignment or to visualize the structural changes in a PDB_REDO optimized structure model. For convenience, and simply to speed up protein structure analyses, we created a set of visualization tools.

Optimized PDB_REDO structure models and their corresponding electron density maps are now directly available within the programs COOT (42) and CCP4mg (43), structure models are also directly available in YASARA (44). COOT additionally shows a list of all significant structural changes. A plugin for PyMOL (http://www.pymol.org/) to show structure models and their electron density is available from the PDB_REDO website. The required maps are generated on-the-fly in the CCP4 (45) format and are also supported by many other programs such as Jmol (46).

Combined PDB and BDB B-factor plots on the BDB website allow the user to rapidly see the corrections made to the PDB B-factors.

On our recently developed web tool pdb-vis (http://www.cmbi.ru.nl/pdb-vis/) secondary structure, symmetry contacts and several accessibility representations are visualized in 2D together with the protein sequence. PDBsum (47) provides many complementary pictures. Several types of 3D structure scenes are also available from pdb-vis, such as scenes of residues that make crystal contacts, or close-ups of metals and bound ligands. Scenes provide a convenient way of highlighting a specific structural feature or local region since the structure, visualization style and viewpoint are stored in a scene file. All scenes can always be inspected with the freely available molecular graphics program YASARA_View (44).

**Figure 2.**
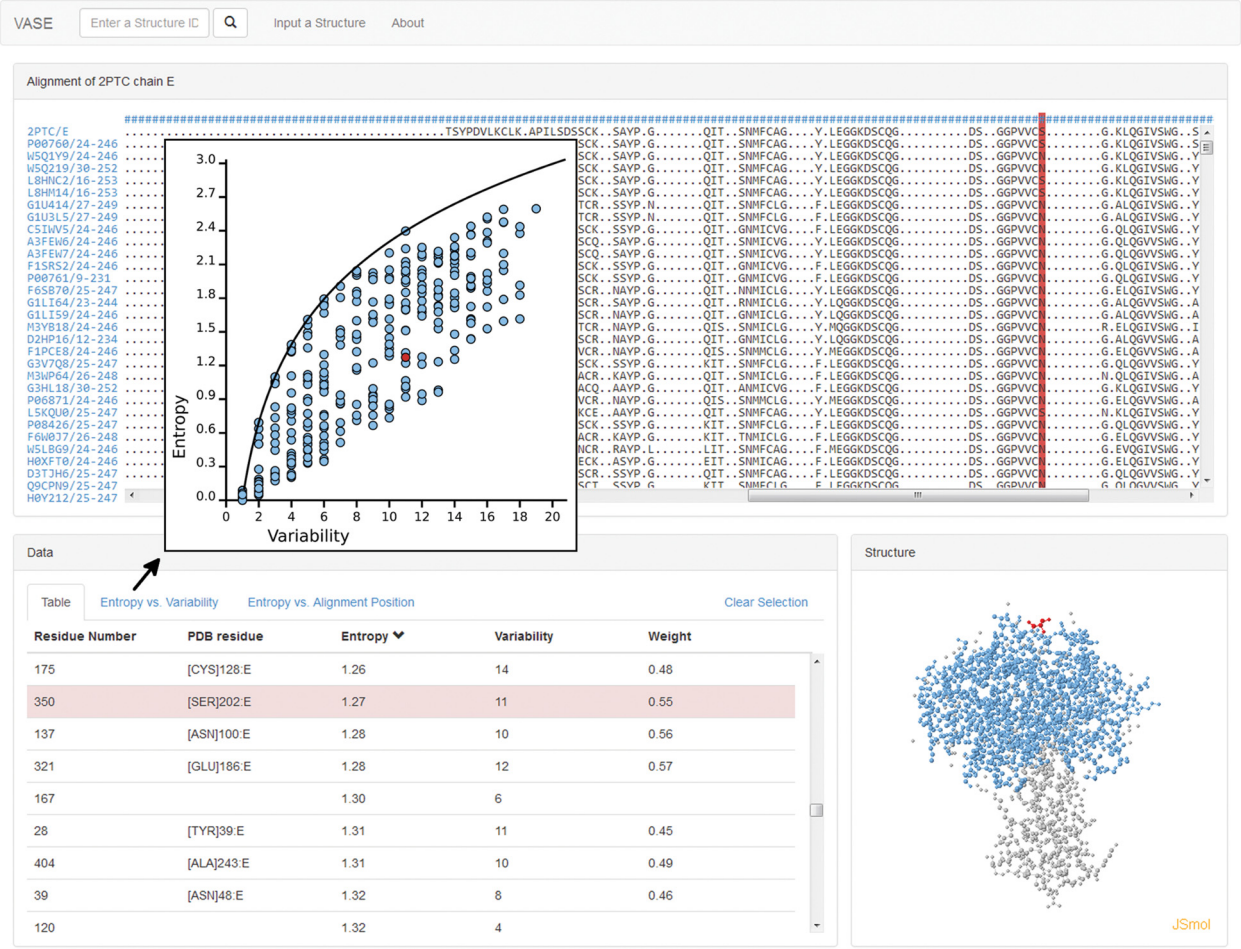
VASE screenshot. A selected position in the HSSP alignment (red column in the top panel) is shown in the structure (bottom right) and in the calculated entropy and variability table (bottom left). The inset shows an entropy-variability plot.

It has long been known that EV analysis of MSAs can elucidate the functional role of residues (48,49). EV values can be calculated from the HSSP alignment and they can be interpreted in a 3D context. We developed the visualization tool VASE (Visualization of Alignments, Structure and Entropy) (50) that connects the three components structure, alignment and entropy/variability in a single browser window. Selected residue positions in the HSSP MSA are color-coded in the 3D structure and *vice versa* (Figure 2). The web interface also shows the EV values (48) for the selected residues in a table or for all residues in an EV plot (Figure 2 inset).

## MATERIALS AND METHODS

DSSP files are produced with the newly written DSSP 2.2.1. HSSP files are produced using HSSP 2.0. PDBFINDER files currently have version 9.0. PDBREPORTs currently are produced with WHAT_CHECK 8.4 (51), but we are planning to release version 11.0 very soon. The PDBREPORT databank will be updated accordingly. The most recent version of PDB_REDO is 5.35. PDB_REDO is under active development and the PDB_REDO databank is continuously being renewed. At the time of writing all files have been created with PDB_REDO version 5.00 or newer which means that all structure models have undergone rebuilding of side chains and flipping of peptide plane orientations when needed (52). BDB files currently are created with version 0.6.5. Most other databanks are produced using the WHAT IF software (53).

## AVAILABILITY

All CMBI's macromolecular structure databanks are freely available and can be accessed in many different ways. The single way of accessing PDB_SELECT lists is through the PDB_SELECT pages. WHY_NOT can be queried for single databank or WHY_NOT entries. Reversely, lists of all absent and annotated entries, present entries, obsolete entries, etc. are available from WHY_NOT. The previously described databanks are indexed by our search system MRS (http://mrs.cmbi.ru.nl/) (54), but the ‘Lists’ and scenes databanks are not. MRS also handles REST or SOAP web service requests. All databanks can be retrieved via rsync and ftp. The rsync protocol allows mirroring entire databanks or a subset of the databank, since all databanks are composed of individual files. Detailed instructions are provided at http://swift.cmbi.ru.nl/gv/facilities/.

The DSSP and HSSP web servers have been renewed and are provided at a single location: http://www.cmbi.ru.nl/xssp/. Existing DSSP and HSSP files can be accessed through this website. Secondary structure assignment of uploaded structure models is also possible via this xssp web server, and additionally via the WHAT IF web servers (http://swift.cmbi.ru.nl/) or the WHAT IF web services (55). A sample script explains the use of the xssp REST API. The ‘check model’ WHAT IF web server section constructs WHAT_CHECK reports from uploaded PDB files. PDB_REDO files can be created using the PDB_REDO web server (24). Some users might prefer creation of databank files on a local workstation. The software for creating BDB, DSSP, HSSP, WHAT IF lists, PDBREPORT, PDB_REDO and YASARA scene databanks is freely available.

## SUPPLEMENTARY DATA

Supplementary Data are available at NAR Online.
